# Harnessing short poly(A)-binding protein-interacting peptides for the suppression of nonsense-mediated mRNA decay

**DOI:** 10.1038/srep37311

**Published:** 2016-11-22

**Authors:** Tobias Fatscher, Niels H. Gehring

**Affiliations:** 1 Institute for Genetics, University of Cologne, Cologne, Germany

## Abstract

Nonsense-mediated mRNA decay (NMD) is a cellular process that eliminates messenger RNA (mRNA) substrates with premature translation termination codons (PTCs). In addition, NMD regulates the expression of a number of physiological mRNAs, for example transcripts containing long 3′ UTRs. Current models implicate the interaction between cytoplasmic poly(A)-binding protein (PABPC1) and translation termination in NMD. Accordingly, PABPC1 present within close proximity of a termination codon antagonizes NMD. Here, we use reporter mRNAs with different NMD-inducing 3′ UTRs to establish a general NMD-inhibiting property of PABPC1. NMD-inhibition is not limited to PABPC1, but can also be achieved by peptides consisting of the PABP-interacting motif 2 (PAM2) of different proteins when recruited to an NMD-inhibiting position of NMD reporter transcripts. The short PAM2 peptides efficiently suppress NMD activated by a long 3′ UTR, an exon-junction complex (EJC) and individual EJC components, and stabilize a PTC-containing β-globin mRNA. In conclusion, our results establish short PABPC1-recruiting peptides as potent but position-dependent inhibitors of mammalian NMD.

Gene expression is an essential and highly regulated process in eukaryotic cells. Several quality control mechanisms are present in the cell to ensure that the correct information is conveyed from DNA level to protein level. One important surveillance mechanism is nonsense-mediated mRNA decay (NMD), which detects and removes erroneous mRNA transcripts such as transcripts harboring premature translation termination codons (PTCs)^1^,^2^,^3^. In mammalian cells PTCs are recognized and NMD is activated if the PTC is located >50 nucleotides upstream of an exon-junction complex (EJC)^4^,^5^, a protein complex deposited on the mRNA during splicing^6^. The central NMD factor UPF1 plays a pivotal role in NMD initiation and serves as an interaction hub for several other NMD factors^7^,^8^,^9^. After the recognition of a PTC, a cascade of events initiates the degradation of the mRNA by the recruitment of the heterodimer SMG5/SMG7 and SMG6^10^.

NMD also plays a role in regulating expression of various physiological mRNAs. However, only some of these endogenous NMD targets contain PTCs, which can potentially result from alternative splicing or upstream open reading frames^11^,^12^. Besides PTC-containing transcripts, several long 3′ UTR containing mRNAs are known to be endogenous NMD targets^12^,^13^,^14^. It has been shown that the 3′ UTR of some endogenous NMD targets are sufficient to induce degradation of the transcript^15^. The mere length of a 3′ UTR has been excluded as the determining factor for degradation of a long 3′ UTR-containing transcript as a study has shown that some long 3′ UTRs are degraded by NMD whereas others are not^16^. The same study suggests instead that the AU-content of a 3′ UTR could potentially mediate the binding of a *cis* factor in close proximity of the termination codon^16^. Recruiting a *cis* factor to the termination codon could possibly suppress activation of NMD. However, it is currently still unknown how a 3′ UTR is marking the transcript for NMD activation or NMD evasion.

It has previously been shown that the cytoplasmic poly(A)-binding protein (PABPC1) can suppress NMD-mediated degradation of a reporter when recruited in close proximity of a termination codon^13^,^17^,^18^,^19^,^20^. Earlier models suggested that the central NMD factor UPF1 competes with PABPC1 for interaction with eukaryotic release factor 3a (eRF3a). If PABPC1 is too far downstream of a terminating ribosome, UPF1 is believed to interact with eRF3a instead. In turn, this is believed to recruit additional NMD factors and will eventually lead to the degradation of the transcript^14^. Two independent studies have shown, however, that the C-terminal domain of PABPC1, which mediates the interaction with eRF3a, is dispensable to suppress NMD of a reporter targeted by NMD^17^,^18^. These findings contradict the competition model between PABPC1 and UPF1 for eRF3a binding as a determining factor for NMD activation. Instead, it has been shown that the interaction between PABPC1 and eukaryotic initiation factor 4 G (eIF4G) is necessary to suppress NMD^17^,^18^. This suggests that an efficient translation termination event could be the responsible determinant for whether NMD is activated or not. The involvement of eIF4G in these findings also suggests a potential role of the closed loop mRNA conformation and ribosome recycling in NMD suppression. Taken together, these findings advocate that the determining factor for NMD suppression might be the recruitment of PABPC1 into close proximity of a terminating ribosome, followed by efficient translation termination and ribosome recycling.

Eukaryotic release factor 3a can interact with PABPC1 via a so-called PABP-interacting motif 2 (PAM2 motif). A region in the PABPC1 C-terminus termed *Mademoiselle* (MLLE) domain has been shown to specifically recognize PAM2 motifs^21^. Essential amino acids (Glutamate, Phenylalanine, Proline) in the PAM2 motif are conserved among several PAM2 motifs from different polypeptides^22^. The PAM2 motif was first discovered in PABP-interacting protein 1 (PAIP1), PAIP2, and eRF3a^23^. More proteins harboring PAM2 motifs have been identified since the discovery of the PAM2 motif^23^,^24^. Different PAM2-containing proteins play roles in different cellular processes. For example, the interaction between PABPC1 and eRF3a has been shown to play a role in polypeptide release and ribosome recycling^22^,^25^,^26^. On the other hand, the PAM2-containing proteins PAN3 and TOB1 play a role in deadenylation. PABPC1 recruits the PAN2-PAN3 deadenylase directly by interacting with PAN3^27^. The other deadenylase, CCR4-NOT, is not directly recruited to the mRNA via PABPC1 but through TOB1 interacting with PABPC1^28^. Furthermore, the PABPC1-interacting protein PAIP2 is a known repressor of translation^29^. It is interesting to note that PABPC1 can interact with a multitude of PAM2-containing proteins with potentially contradictory functions. It is currently unknown how the interaction between PABPC1 and the many PAM2-containing proteins is regulated.

Many genetic disorders are caused by nonsense mutations, for which NMD acts as a modifier and eliminates truncated proteins with potential deleterious effects^30^,^31^,^32^. However, not all truncated proteins have a negative effect. In some instances, increased amounts of a truncated protein can lead to a less severe form of a disease^33^. Suppressing NMD could be favorable in medical conditions where the presence of a truncated protein would be advantageous over the complete absence of the protein. Elevated levels of full length proteins could be achieved by a combined treatment with drugs that induce translational read-through^32^.

Earlier studies reported that recruiting PABPC1 into close proximity of a termination codon suppresses NMD. Here, we investigated different methods of NMD suppression via PABPC1 recruitment. We find that the recruitment of PABPC1 by PAM2 peptides inhibits NMD of reporter transcripts. These results open up new ways of NMD suppression by small biologically active peptides.

## Results

Translation and efficient translation termination play a pivotal role in gene expression and the lifespan of an mRNA. PABPC1 is an essential factor during this process, promoting mRNA circularization via its interaction with eIF4G, suppressing NMD at normal termination codons, and facilitating ribosome recycling^20^,^34^,^35^,^36^. A revised model of how PABPC1 can possibly act to suppress NMD when in close proximity of a termination codon was previously proposed^17^,^18^,^19^. Here, we further investigate mechanisms of NMD suppression by PABPC1.

### PABPC1 and eRF3a suppress NMD of additional long 3′ UTR containing reporter mRNAs

**Figure 1:**
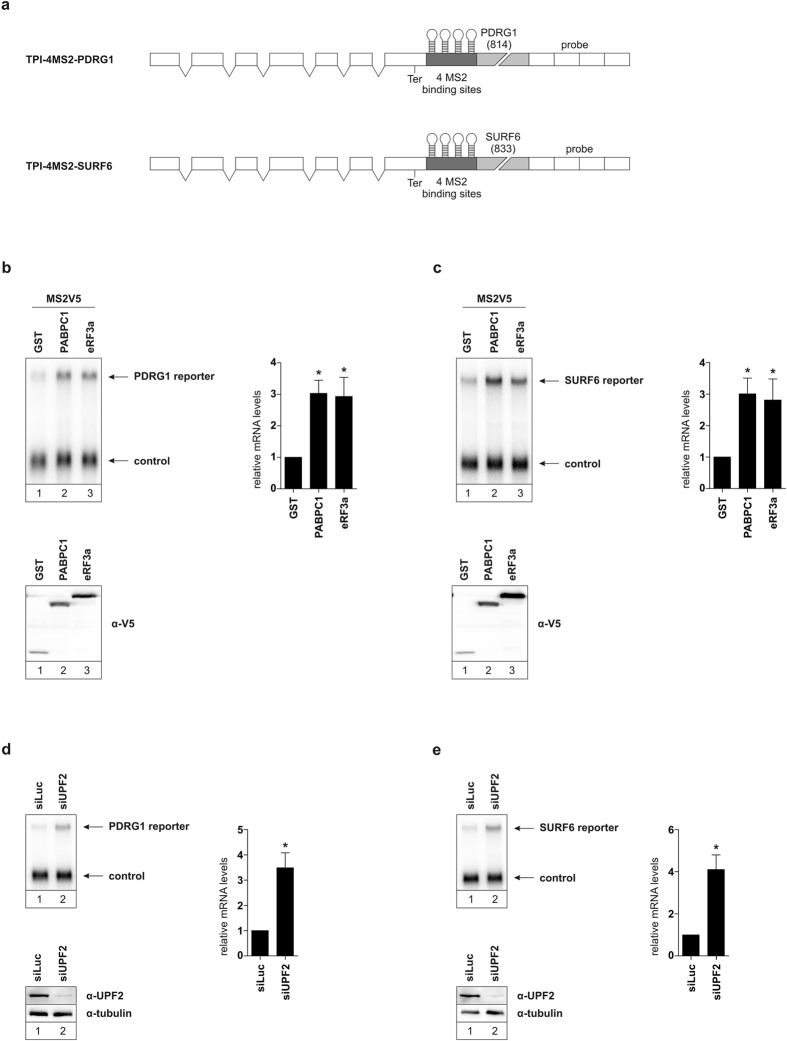
Identifying two additional long 3′ UTR-containing reporter constructs stabilized by PABPC1 and eRF3a tethering (**a**) Schematic representation of the two triosephosphate isomerase (TPI) tethering reporter constructs containing either the 3′ UTR of PDRG1 (814 nts) or SURF6 (833 nts). (**b** and **c**) Northern blot analysis of total RNA extracted from HeLa cells transfected with plasmids expressing the indicated TPI reporter mRNA and MS2V5 fusion proteins. β-globin wildtype was co-transfected as a control. Protein expression was detected by immunoblotting with an α-V5 antibody. mRNA levels were normalized to MS2V5-GST. Black bars represent the mean values of mRNA levels ± standard deviation upon tethering MS2V5-GST, MS2V5-PABPC1, or MS2V5-eRF3a (n = 4). P values were calculated relative to MS2V5-GST conditions using the paired two-tailed Student’s t-test. * Depicts a P value of P < 0.05. (**d** and **e**) Northern blot analysis of total RNA extracted from HeLa cells transfected with plasmids expressing the indicated TPI reporter mRNA and siRNAs for either luciferase or UPF2. β-globin wildtype was co-transfected as a control. Expression of UPF2 was detected by immunoblotting with an α-UPF2 antibody. mRNA levels were normalized to siLuc condition. Black bars represent the mean values of mRNA levels ± standard deviation upon knockdown of UPF2 (n = 3). P values were calculated relative to siLuc conditions using the paired two-tailed Student’s t-test. * Depicts a P value of P < 0.05.

We have previously reported that both PABPC1 and eRF3a are able to suppress NMD of a reporter carrying the long 3′ UTR of SMG5^17^. We next aimed to generalize this observation, since our previous findings were based on a single reporter mRNA with the SMG5 3′ UTR. To this end, we constructed two tethering reporters containing the 3′ UTRs of PDRG1 and SURF6 downstream of 4MS2 binding sites (Fig. 1a). Both, PDRG1 and SURF6 were identified by a transcriptome-wide screen in our laboratory as endogenous NMD substrates (unpublished data Gehring lab). As expected, both PABPC1 and eRF3a were able to antagonize degradation via NMD of both reporter constructs and increased the mRNA levels about 3-fold compared to a negative GST-control (Fig. 1b and c). Hence, NMD suppression by both PABPC1 and eRF3a is a general phenomenon that can act on different substrate mRNAs targeted by NMD due to their 3′ UTR. To show that PDRG1 and SURF6 reporters are targeted by NMD, we knocked down the NMD factor UPF2 in cells transfected with each reporter. This led to an increase in PDRG1 and SURF6 reporter mRNA levels of about 3.5- and 4-fold compared to a luciferase control knockdown, respectively (Fig. 1d and e).

### Suppressing NMD by indirectly recruiting PABPC1

**Figure 2:**
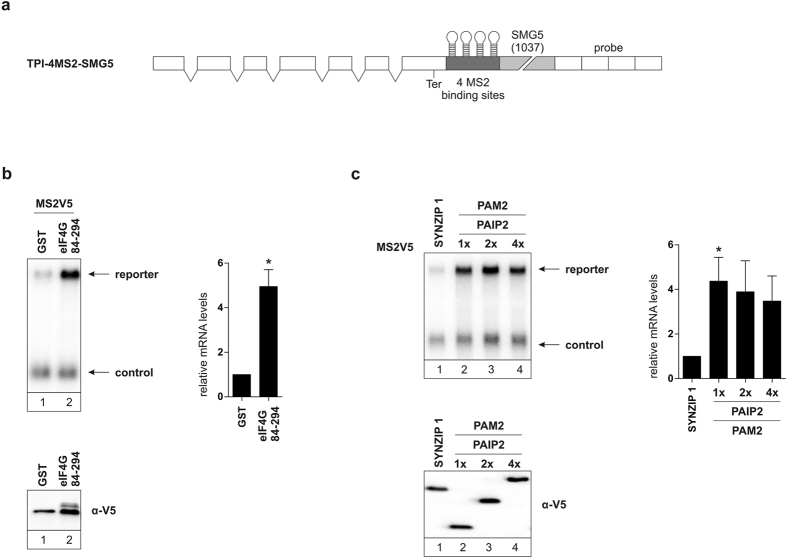
NMD suppression by short PABPC1-recruiting proteins and peptides. (**a**) Schematic representation of the TPI reporter containing a shortened form of the SMG5 3′ UTR (1037 nts). (**b** and **c**) Northern blot analysis of total RNA extracted from HeLa cells. HeLa cells were transfected with MS2V5-GST and MS2V5-eIF4G 84-294 (**b**), and MS2V5-SYNZIP 1, MS2V5-PAIP2 PAM2 1x/2x/4x, β-globin wildtype was used as a control mRNA. Protein expression was detected by immunoblotting with an α-V5 antibody (**b** and **c**). Black bars represent the mean values of mRNA levels ± standard deviation upon tethering MS2V5-GST and MS2V5-eIF4G 84-294 (**b**) (n = 4), and MS2V5-SYNZIP 1, MS2V5-PAIP2 PAM2 1x/2x/4x, and MS2V5-PAIP2 PAM2 mut 1x/2x/4x (**c**) (n = 3). mRNA levels were normalized to MS2V5-GST (**b**) and MS2V5-SYNZIP 1 (**c**). P values were calculated relative to MS2V5-GST or MS2V5-SYNZIP 1 conditions using the paired two-tailed Student’s t-test. * Depicts a P value of P < 0.05.

It has previously been shown that eIF4G is able to suppress NMD, when tethered in close proximity of a termination codon^17^,^18^, even when lacking the PABPC1 interaction site. Here, we made use of an eIF4G deletion mutant (eIF4G 84–295), which only contains the interaction site for PABPC1. The short form of eIF4G was still able to suppress NMD of a reporter containing the SMG5 3′ UTR, which could be seen by a 5-fold increase in reporter mRNA (Fig. 2b). This result suggests that eIF4G is able to suppress NMD with no other factors involved, but simply by recruiting PABPC1. We previously showed that eIF4G is able to suppress NMD without the need for PABPC1^17^. In contrast, we show here that NMD can be suppressed by simply recruiting PABPC1 to an NMD-targeted reporter via a short version of eIF4G that does not interact with any other protein but PABPC1. The fact that the deletion mutant of eIF4G, containing only the PABPC1 interaction region, is able to suppress NMD, suggests that recruiting PABPC1 via a short peptide is sufficient for NMD inhibition.

PABPC1 contains a well-conserved 70 amino acid domain termed MLLE in its C-terminal region^23^. It has been shown that this MLLE domain specifically recognizes the small peptide sequence called PAM2 motif^21^. Initially, three different proteins were identified carrying a PAM2 motif: PAIP1 (PABP-interacting protein 1), PAIP2, and eRF3^23^. To assess the NMD suppression potential of recruiting PABPC1 via short peptides, we made use of the isolated PAM2 motif of PAIP2 to recruit PABPC1 to the reporter mRNA (Fig. 2c). Interestingly, recruiting PABPC1 via the PAIP2 PAM2 motif suppresses NMD and results in increased reporter mRNA levels of about 4-fold (Fig. 2c, lane 2). An MS2V5-tagged version of SYNZIP 1, a short artificial peptide with no inherent function, was used as a negative control (Fig. 2c, lane 1)^37^. To assess whether PABPC1 crowding near a termination codon has an effect on NMD suppression, we cloned two and four PAIP2 PAM2 motifs separated by short spacers in succession and tethered these to the reporter mRNA. Tethering MS2V5-tagged PAIP2 PAM2 2x and PAIP2 PAM2 4x to the reporter did not lead to an additional increase in mRNA reporter levels compared to MS2V5-PAIP2 PAM2 (Fig. 2c, compare lane 2 to lanes 3 and 4). This shows that recruiting more PABPC1 via multiple PAM2 motifs in succession to an NMD-inhibiting position on a reporter mRNA does not result in an increased NMD suppression compared to a single PAIP2 PAM2 motif. Instead we suggest that tethering a PAM2 motif just downstream of a termination codon leads to the folding back of the poly(A) tail by PAM2 interacting with poly(A)-bound PABPC1. This would also explain the lack of increased NMD suppression by employing multiple PAM2 motifs in succession, as one PAM2 motif is enough to interact with and fold back poly(A)-bound PABPC1.

### Tethering PAM2 peptides to an NMD-targeted reporter suppresses degradation

**Figure 3:**
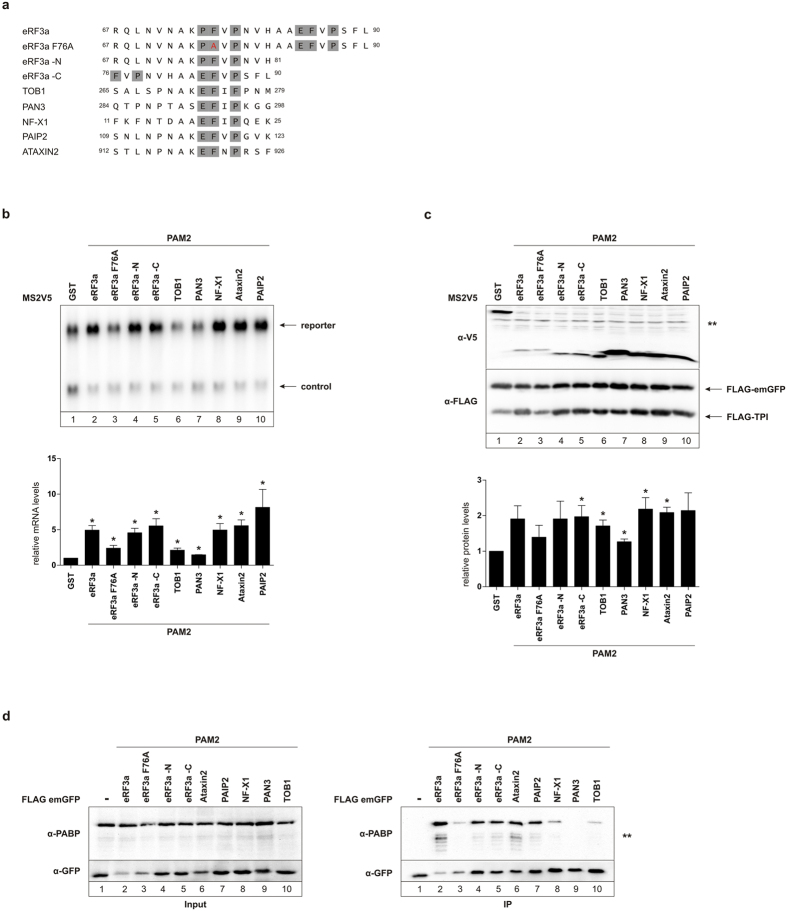
PAM2 peptides are able to antagonize NMD of a long 3′ UTR reporter mRNA. (**a**) Schematic representation of the amino acid make up of several PAM2 motifs from different proteins. Gray boxes indicate residues important for interaction with MLLE domain of PABPC1. Red letter shows a point mutation of eRF3a PAM2 motif abrogating interaction with PABPC1. The numbers indicate the beginning and end amino acids of the PAM2 motifs. (**b**) Northern blot analysis of total RNA with transfected MS2V5-tagged PAM2 motifs. N-terminally FLAG-tagged TPI-4MS2-SMG5 was used as a reporter mRNA. β-globin wildtype was used as a control RNA. (**c**) Protein expression of the MS2V5-tagged PAM2 motifs was detected with an α-V5 antibody. FLAGemGFP was transfected as an expression control. FLAG-tagged TPI was used to determine relative protein levels. ** Represents an unspecific band. (**b** and **c**) Quantification of mRNA levels (**b**) and TPI protein expression (**c**). Black bars represent the mean values of mRNA levels (**b**) and relative protein levels (**c**) upon tethering PAM2 motifs to the reporter mRNA (n = 3). P values were calculated relative to MS2V5-GST conditions using the paired two-tailed Student’s t-test. * Depicts a P value of P < 0.05. (**d**) Co-immunoprecipitation of FLAGemGFP-tagged PAM2 motifs with endogenous PABPC1. Input is shown on the left and immunoprecipitation on the right. GFP antibody was used to detect PAM2 motif expression and α-PABP antibody to detect endogenous PABPC1 co-precipitated with PAM2 motifs. ** Represents an unspecific band.

So far we have established that the PAM2 motif of PAIP2 is able to suppress NMD of a long 3′ UTR reporter (Fig. 2). Other PAM2-containing proteins have been identified based on sequence homology^22^. Interestingly, eRF3 is different compared to other PAM2-containing proteins since it carries two overlapping PAM2 motifs^21^. Since PABPC1 strongly interacts with proteins harboring a PAM2 motif, we tethered the isolated PAM2 motifs of several proteins to recruit PABPC1 to an NMD-targeted reporter (Fig. 3a). Tethering the MS2V5-tagged overlapping N- and C- terminal eRF3a PAM2 motif to the previously used FLAG-TPI-4MS2-SMG5 reporter increased the levels of the reporter mRNA about 5-fold (Fig. 3b, lane 2)^17^. As a negative control, we used a point mutation of the PAM2 motif of eRF3a (eRF3a F76A) known to reduce the binding affinity between eRF3a and PABPC1^22^,^38^,^39^. Tethering MS2V5-eRF3a F76A PAM2 to the reporter mRNA had a much smaller effect compared to eRF3a containing both PAM2 motifs (Fig. 3b, lane 3). Tethering the N- and C-terminal PAM2 motifs of eRF3a separately to the NMD-targeted reporter increases the reporter mRNA levels also about 5-fold (Fig. 3b, lanes 4 and 5). The PAM2 motifs of NF-X1, Ataxin2, and PAIP2 also increased the reporter mRNA levels by 5- to 8-fold upon tethering (Fig. 3b, lanes 8, 9, and 10). Surprisingly, tethering MS2V5-tagged TOB1 and PAN3 PAM2 motifs to the reporter only led to a small increase of the reporter mRNA (Fig. 3b, lanes 6 and 7).

The poly(A) tail and PABPC1 are considered to determine translation efficiency in eukaryotes^40^. Given the strong increase in mRNA levels upon tethering PAM2 motifs to the reporter we wanted to exclude that NMD is indirectly suppressed due to the inhibition of translation. To this end we employed the N-terminal FLAG-tag of the TPI reporter to quantify protein expression compared to FLAGemGFP, which was transfected as control for protein expression (Fig. 3c, lower panel). The protein levels upon tethering were increased approximately 2-fold for the eRF3a, as well as the NF-X1, Ataxin2, and PAIP2 PAM2 motifs (Fig. 3c). In contrast, tethering of the less active eRF3a F76A PAM2 motif as well as PAM2 of TOB1 and PAN3 led to a slightly less increase in protein expression from the reporter mRNA (Fig. 3c). The implication of this smaller increase in protein abundance remains unclear.

It is surprising that some PAM2 peptides are very efficient inhibitors of NMD, while others show only very little activity. We hypothesized that differences in the interaction of the PAM2 peptide with PABPC1 are causing the observed differences. To this end, we expressed FLAGemGFP-tagged PAM2 motifs and subjected them to a FLAG pulldown. We used an antibody against endogenous PABPC1 to check for interactions between PAM2 motifs and PABPC1. The strongest interaction was observed for the overlapping eRF3a PAM2 motifs (Fig. 3d, lane 2, right panel), although the eRF3a PAM2 motif consistently exhibited very low expression levels. As expected, eRF3a F76A PAM2 pulled out significantly less endogenous PABPC1 (Fig. 3d, lane 3, right panel). The residual interaction between eRF3a F76A and endogenous PABPC1 can be attributed to the second Phenylalanine still present in this mutant. Furthermore, strong interactions between eRF3a -N PAM2, eRF3a -C PAM2, Ataxin2 PAM2, PAIP2 PAM2, and endogenous PABPC1 were observed (Fig. 3d, lanes 4, 5, 6, and 7, right panel). NF-X1 PAM2 and TOB1 PAM2 pulled out less endogenous PABPC1, whereas PAN3 PAM2 did not interact with endogenous PABPC1 at all (Fig. 3d, lanes 8, 10, and 9, right panel). These results explain the inefficient NMD inhibition by the PAM2 motifs of PAN3, and the reduced NMD suppression by TOB1 and the negative control eRF3a F76A PAM2. It is surprising that some PAM2 motifs are unable to interact with PABPC1 even though the essential amino acids for the interaction with PABPC1 are present. Interestingly, PAIP2 PAM2 was able to stabilize the reporter mRNA more strongly than the other PAM2 motifs. The reason behind these different interaction and stabilization patterns is currently unknown.

### PAM2-mediated NMD suppression is position dependent

**Figure 4:**
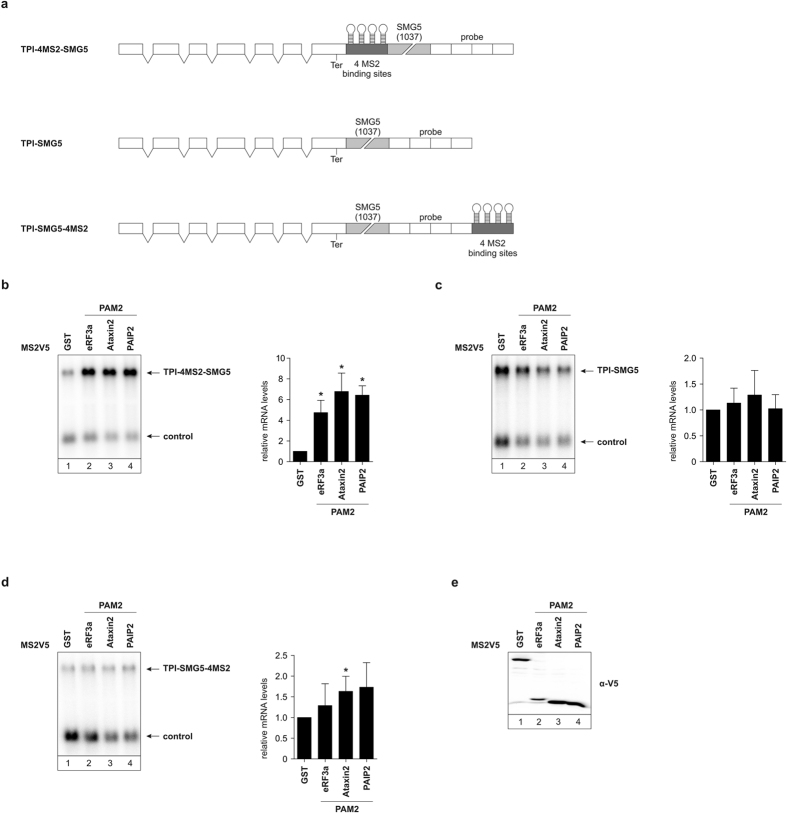
NMD inhibition by PAM2 peptides is specific and position-dependent. (**a**) Schematic representation of TPI tethering reporters used in these assays. (**b,c**, and **d**) Northern blot analysis of total RNA extracted from HeLa cells transfected with plasmids expressing the indicated TPI reporter mRNA and MS2V5 fusion proteins. β-globin wildtype was co-transfected as a control. Black bars represent the mean values of mRNA levels ± standard deviation upon tethering MS2V5-GST, MS2V5-eRF3a PAM2, MS2V5-Ataxin2 PAM2, or MS2V5-PAIP2 PAM2 (n = 4 for **b**, n = 5 for **c**–**e**). mRNA levels were normalized to MS2V5-GST. P values were calculated relative to MS2V5-GST conditions using the paired two-tailed Student’s t-test. * Depicts a P value of P < 0.05. (**e**) Protein expression of the MS2V5-tagged PAM2 motifs was detected with an α-V5 antibody.

Next, we made use of several different control reporter constructs to test the specificity of tethering different PAM2 motifs and to exclude non-specific *trans* effects. To this end we used a reporter lacking any MS2-binding sites and a reporter carrying MS2-binding sites downstream of the SMG5 3′ UTR (Fig. 4a). We used the eRF3a, Ataxin2, and PAIP2 PAM2 motifs for this assay since these have shown the strongest NMD-antagonizing effect of the PAM2 motifs studied here. First, we tethered the three PAM2 motifs separately to the TPI-4MS2-SMG as a positive control, resulting in an increase in mRNA reporter abundance (Fig. 4b). Tethering the PAM2 motifs to the reporter without any MS2-binding sites or with the MS2 sites downstream of the SMG5 3′ UTR did not lead to an increase in mRNA abundance (Fig. 4c and d) indicating the specificity of the tethering assay. Western blotting showed the expression of all constructs to be similar and excludes a lack of expression as a possibility for failed reporter mRNA stabilization (Fig. 4e).

### PAM2 peptides efficiently antagonize EJC-mediated NMD

**Figure 5:**
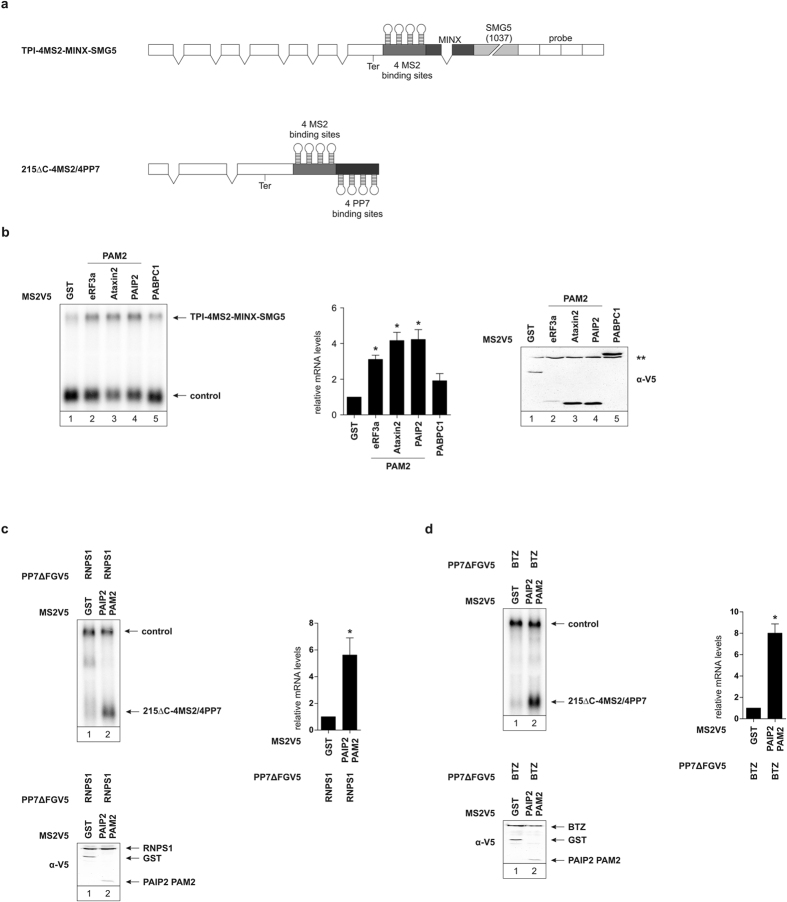
Suppression of EJC-mediated NMD via tethered PAM2 peptides. (**a**) Schematic representation of the TPI tethering reporter and the β-globin double tethering reporter containing 4MS2/4PP7 binding sites used in these assays. (**b,c**, and **d**) Northern blot analysis of total RNA extracted from HeLa cells transfected with plasmids expressing the indicated TPI (**b**) or β-globin (**c** and **d**) reporter mRNA and MS2V5 (**b**) and MS2V5/PP7ΔFGV5 (**c** and **d**) fusion proteins. β-globin wildtype (**b**) and lacZ (**c** and **d**) were co-transfected as a control. Black bars represent the mean values of mRNA levels ± standard deviation upon tethering MS2V5- (**b**) and MS2V5/PP7ΔFGV5-fusion (**c** and **d**) proteins (n = 3). mRNA levels were normalized to MS2V5-GST (**b**), MS2V5-GST/PP7ΔFGV5-RNPS1 (**c**), and MS2V5-GST/PP7ΔFGV5-BTZ (**d**). P values were calculated relative to MS2V5-GST conditions using the paired two-tailed Student’s t-test. * Depicts a P value of P < 0.05. (**b,c**, and **d**) Protein expression was detected by immunoblotting with an α-V5 antibody. ** Represents an unspecific band (**b**).

So far we have shown that PAM2 motifs are able to efficiently suppress NMD activated by the SMG5 3′ UTR by recruiting PABPC1 into close proximity of the termination codon. However, many NMD substrates are not targeted due to a 3′ UTR, but are activated by the splicing-dependent EJC, which is deposited at exon-exon junctions. To address whether PAM2 mediated NMD suppression can also suppress NMD activated by an EJC, we next made use of a TPI reporter carrying an intron (MINX) downstream of the 4MS2-binding sites and upstream of the SMG5 3′ UTR (Fig. 5a). During RNA processing the intron will be spliced out of the transcript and an EJC is deposited downstream of the 4MS2 sites. We previously reported that tethering PABPC1 to this reporter does not lead to an increase in mRNA abundance^17^. Here, we observed a 5 to 6-fold increase in reporter mRNA abundance upon tethering eRF3a, Ataxin2, and PAIP2 PAM2 motifs upstream of the MINX intron (Fig. 5b). Tethering MS2V5-PABPC1 to this reporter led to significantly less stabilized reporter mRNA levels compared to the PAM2 motifs (Fig. 5b, compare lane 5 to lanes 2–4). These results establish that recruiting PABPC1 through a small PAM2 motif leads to significant NMD inhibition.

The reporter that we have used before (Fig. 5a) contains both an intron and a long 3′ UTR. To directly test the ability of PAM2 peptides to suppress NMD by an EJC, we chose to activate NMD by tethering of two EJC-factors: RNPS1 and Barentsz (BTZ or MLN51). RNPS1 was first identified as a general splicing activator and is known to initiate NMD when tethered to a reporter^41^,^42^. Similarly, BTZ, part of the EJC core complex, has also previously been shown to initiate NMD of a reporter upon tethering to the construct^43^. For this experiment, we constructed a double tethering reporter harboring 4MS2 binding sites upstream of 4PP7 binding sites (Fig. 5a). This double tethering reporter allows us to tether two different fusion proteins in a specific spatial orientation. When tethering the PAM2 motif of PAIP2 upstream of RNPS1 we observed an approximately 6-fold increase of reporter mRNA levels compared to tethering RNPS1 downstream of GST (Fig. 5c). Likewise, NMD initiated by tethering BTZ was suppressed by tethering the PAM2 motif of PAIP2 upstream of BTZ (Fig. 5d). Tethering PAIP2 PAM2 in this spatial orientation leads to an 8-fold increase in reporter mRNA levels compared to BTZ tethered downstream of GST (Fig. 5d). These results indicate that the PAM2 motif of PAIP2 is not only able to suppress 3′ UTR-mediated but also EJC-mediated NMD. In combination, these results are a strong indication for an NMD suppression ability of PAM2 motifs when tethered upstream of an EJC.

### Recruiting PABPC1 suppresses NMD of a PTC-containing mRNA

**Figure 6:**
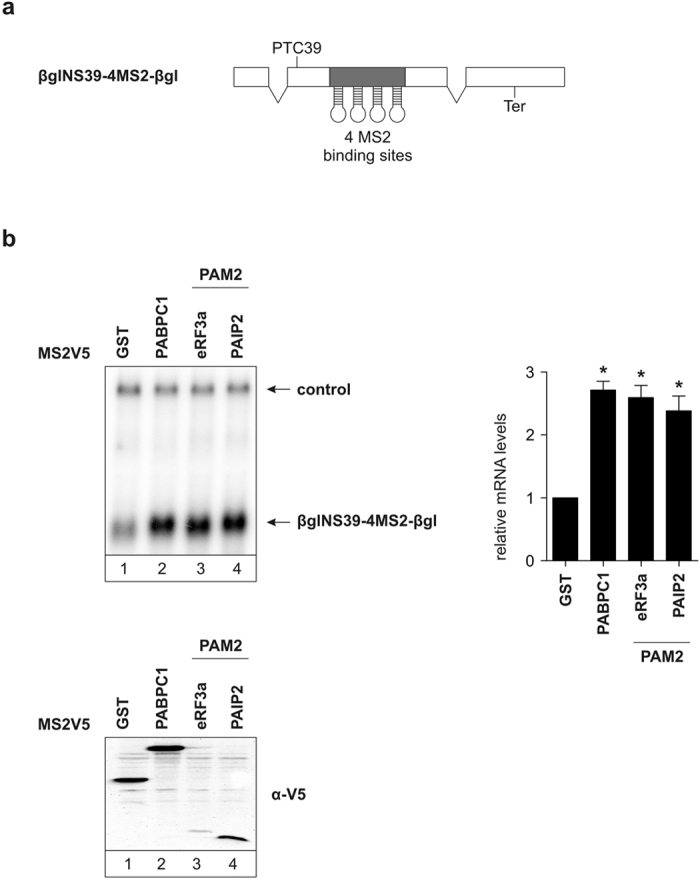
NMD inhibition of a PTC-carrying reporter. (**a**) Schematic representation of the β-globin PTC39 tethering reporter containing 4MS2 binding sites directly downstream of the PTC. (**b**) Northern blot analysis of total RNA extracted from HeLa cells transfected with plasmids expressing β-globin PTC39 reporter mRNA and MS2V5-fusion proteins. LacZ was used as a control. Black bars represent the mean values of mRNA levels ± standard deviation upon tethering MS2V5-fusion proteins (n = 3). mRNA levels were normalized to MS2V5-GST. P values were calculated relative to MS2V5-GST conditions using the paired two-tailed Student’s t-test. * Depicts a P value of P < 0.05. Protein expression was detected by immunoblotting with an α-V5 antibody.

As we have so far shown that PABPC1 recruited via a tethered PAM2 motif can strongly suppress NMD either activated by a long 3′ UTR or a downstream EJC, we next wanted to examine the ability of PAM2 peptides to suppress NMD of a PTC-containing mRNA. Human β-globin carrying a PTC at position 39 has been identified in patients suffering from β-thalassemia and is a well-known NMD target^4^,^44^,^45^. We used a reporter with MS2 binding sites inserted downstream of the PTC at position 39. The remainder of the β-globin DNA was cloned after the MS2 binding sites (Fig. 6a). This reporter construct allows us to tether an NMD antagonizing factor in a close to natural setting. This reporter carries an intron downstream of the PTC, which results in an EJC deposited downstream of the terminating ribosome, thereby initiating NMD. Tethering MS2V5-PABPC1 to this reporter leads to an increase in mRNA reporter abundance by about three fold (Fig. 6b, lane 2). The same magnitude of stabilization is observed when tethering the PAM2 motifs of eRF3a and PAIP2 to the reporter (Fig. 6b, lanes 3 and 4). Taken together these results establish that PAM2 peptides have the capability to suppress NMD of an endogenous NMD target when bound at a position downstream of the termination codon and upstream of the next NMD-activating EJC.

## Discussion

Mammalian NMD degrades mRNAs that carry an EJC downstream of the termination codon or that contain a long 3′ UTR. It has been previously demonstrated that the physical distance between the termination codon and the poly(A)-tail is important to discriminate between a proper and an aberrant termination event^13^. Different mechanisms of NMD evasion have been previously described for NMD substrates. Firstly, a 200nt A/U-rich region immediately downstream of a termination codon has been shown to be responsible for NMD suppression^16^. This region could potentially be bound by an as of yet unknown protein or proteins, which are either able to block NMD by disrupting the interaction with eRF3a and UPF1, or by interacting with PABPC1 directly. Recently, PTBP1 was identified as such a protein that binds to some mRNAs with a long 3′ UTR just downstream of the termination codon, thereby blocking NMD^46^. PTBP1 binds preferentially to CU-rich regions and a single PTBP1 protein has multiple RNA binding sites. This way it is conceivable that PTBP1 could compact the 3′ UTR by binding stretches of the 3′ UTR further apart. By doing so PABPC1 would be brought into closer proximity of the terminating ribosome, which could lead to NMD suppression. It has also been previously reported that tethering PABPC1 or inserting a stretch of adenosines immediately downstream of a PTC suppresses NMD by reducing the binding of UPF1 to the mRNA^47^. Hence, PABPC1 may be involved in the active dissociation of UPF1 from stable, non-target mRNAs.

The strongest NMD antagonizing effect we observed here resulted from tethering PAM2 peptides to a reporter construct targeted by NMD (Fig. 3). Specifically, the 15 amino acid PAM2 motif of PAIP2 showcased the strongest suppression of NMD induced by the SMG5 3′ UTR. The results obtained by tethering PAM2 motifs to the NMD reporter were surprising. For instance, recruiting PABPC1 via the PAIP2 PAM2 peptide to the SMG5 3′ UTR reporter resulted in a 3-fold higher mRNA abundance compared to what we observed in our previous work when tethering PABPC1 directly^17^. We have currently no explanation as to why this difference in NMD suppression strength is occurring. Besides PABPC1, the PAM2 motif is known to only interact with EDD, the human homolog of the *Drosophila* tumor suppressor gene *hyperplastic discs* (hyd)^48^. EDD has been shown to share a 56% similarity of the PABP C-terminal domain and is able to interact with PAIP1^49^. However, to our knowledge EDD is not implicated in the NMD mechanism and therefore we postulate that the NMD inhibiting effects of PAM2 motifs is mediated by their binding to PABPC1. A possible theory as to why we observe a much stronger stabilization by tethered PAM2 motifs could be that the MLLE domain of PABPC1 is blocked when PABPC1 is recruited to the reporter via a tethered PAM2 motif. In principle, when PABPC1 is tethered directly it could theoretically still interact with other PAM2 carrying proteins, which might regulate its NMD suppression ability. Obstructing the MLLE domain of PABPC1 when it is recruited via a PAM2 motif would remove the possibility of other PAM2-carrying proteins interacting with PABPC1 and possibly reducing its NMD-inhibiting effect. This hypothesis would also be in line with the previous observation that PABPC1 does not require its MLLE domain for NMD suppression^17^,^18^. Our result that tethering of multiple PAM2 motifs does not enhance NMD suppression suggests an alternative explanation for the remarkable efficiency of PAM2 peptides. Because a single PAM2 suffices to suppress NMD, a tethered PAM2 peptide may fold back the poly(A) tail by interacting with poly(A)-bound PABPC1. A similar fold-back may be exerted by the PABPC1-interacting domain of eIF4G, which is also a potent inhibitor of NMD in our hands. Folding back the poly(A) tail has been shown to strongly suppress NMD^13^, but its mechanism has not been studied in detail. Nonetheless, it is likely that NMD inhibition by the poly(A)-PABPC1 entity involves the interaction of PABPC1 with eIF4G, which has been suggested to promote ribosome recycling.

It was also surprising to see that not all PAM2 motifs have the same capability to antagonize NMD. For example, the TOB1 and PAN3 PAM2 motifs do not stabilize the SMG5 3′ UTR reporter construct (Fig. 3b). The most likely explanation for this is the lack of interaction of these two PAM2 motifs with endogenous PABPC1 (Fig. 3d). This is puzzling as the 15 amino acid PAM2 peptides of these two proteins contain all three of the crucial amino acids of a PAM2 motif required for the interaction with PABPC1. More work will be required to understand the determinants of the interaction between PAM2 peptides and PABPC1 under cellular conditions.

Many inherited diseases are caused by frameshift or nonsense mutations leading to transcripts carrying a PTC. These transcripts activate NMD due to one or more EJCs downstream of the termination codon. Here we show that inhibiting NMD by tethering of a PAM2 motif is not limited to 3′ UTR activated NMD. We have shown here that PAM2 peptides are able to suppress NMD activated by either an EJC deposited during splicing or by single EJC components (Fig. 5). Furthermore, tethering PAM2 peptides downstream of PTC39 in the β-globin mRNA inhibited NMD of this well-established NMD reporter. The fact that a small PAM2 motif can suppress EJC-mediated NMD is surprising as we previously reported that full length PABPC1 is unable to suppress EJC NMD when tethered upstream of an EJC^17^. It will be an important task for future studies to elucidate the differences between EJC- and 3′ UTR-activated NMD and their suppression by PABPC1 and PAM2 peptides.

We have established tethering of PAM2 motifs as a new means for specifically antagonizing the degradation of NMD substrates. Recruiting PABPC1 in close proximity of a termination codon via a small PAM2 peptide suffices to inhibit both 3′ UTR- and EJC-mediated NMD. Due to their small size, PAM2 peptides are ideal for the large-scale synthesis of biologically active components and their activity may be further enhanced by synthetic modifications and/or mutagenesis. So far, we have only demonstrated the activity of PAM2 motifs by tethered function analysis. However, PAM2 peptides also may be combined with sequence-specific RNA-binding molecules, such as Pumilio homology domains^50^,^51^, CRISPR-Cas9^52^,^53^ or antisense oligonucleotides^54^ to suppress NMD independent of tethering. Applied in combination with read-through drugs, PAM2 peptides may also enable to restore the expression of nonsense-mutated genes. Future studies will help to exploit the remarkable NMD-inhibiting potential of PAM2 peptides in therapeutic applications.

## Experimental Procedures

### Plasmids constructs

Plasmid constructs β-globin, pCI-FLAGemGFP, pCI-MS2V5, pCI-mVenus, pCI-TPI and expression vector for PABPC1 and eIF4G were described previously^55^,^56^,^57^,^58^,^59^. The modification of pCI-TPI with four copies of binding sites for the heterologous probe used in northern blot analysis, as well as the insertion of the SMG5 3′ UTR and 4MS2 binding sites in the 3′ UTR of TPI were described elsewhere^15^. Using the same cloning strategy three more reporters were created: the 3′ UTR of PDRG1 and SURF6 were introduced into the TPI vector, and 4PP7- and 4MS2-binding sites enclosing the SMG5 3′ UTR were cloned into the TPI vector. The β-globin-PTC39–4MS2-β-globin reporter was constructed by inserting 4MS2-binding sites at position 112 nucleotides into the second exon of β-globin. The remainder of β-globin was inserted downstream of these 4MS2-binding sites. The MINX-containing reporters, as well as the SMG5 3′ UTR reporter containing alternate termination codon sites were described previously^17^. Deletion and point mutants of eRF3a and eIF4G were generated by site-directed mutagenesis. The PAM2 containing mutants were generated by PCR and cloned in the designated expression vector. All plasmid constructs were verified by sequencing.

### PAM2 plasmid construction

PAM2 motifs were cloned by PCR using two overlapping primers. The finished PCR products were digested with XhoI and NotI, followed by ligation into either pCI-MS2V5 XhoI/NotI or pCI-FLAGemGFP XhoI/NotI.

### Plasmid transfections

HeLa cells were grown in 6-well plates or 6 cm dishes at concentrations of 240,000 cells/ml and 650,000 cells/ml, respectively. Transfections were done by calcium phosphate precipitation. For tethering and overexpression experiments 0.3 μg of an mVenus expression plasmid, 0.5 μg control plasmid (β-globin), 2.0 μg plasmid encoding for reporter mRNA, and 0.8 μg of MS2V5-, PP7∆FGV5-, and λNV5-expression plasmids were used in the transfection mix. For co-immunoprecipitation experiments 1.5 μg FLAGemGFP-expression plasmids and 1.0 μg Salmon DNA were added to a transfection mix. For translation efficiency experiments we included 1.5 μg FLAGemGFP as control and 2.0 μg FLAG-TPI-SMG5-4H as reporter in the transfection mix.

### siRNA transfections

Delivery of siRNAs into cells was performed by reverse transfecting 60 pmol siRNA per 2 × 10^5^ cells using 2.5 μl Lipofectamine RNAiMAX (Thermo Fisher). The following siRNA target sequences were used: luciferase (5′-CGTACGCGGAATACTTCGA-3′) and UPF2 (5′-CGTTGTGGATGGAGTGTTA-3′).

### RNA extraction and northern blotting

Total RNA was extracted with peqGOLD TriFast lysis reagent (Peqlab) and analyzed by northern blotting as described^56^. Signals were quantified using a Typhoon FLA 7000 (GE Healthcare).

### Immunoblot analysis

SDS-polyacrylamide gel electrophoresis and immunoblot analysis was performed using protein samples derived from peqGOLD Trifast extractions. The antibody against FLAG (F7425; 1:3000 dilution) was from Sigma, the antibody against V5 (18870; 1:3000 dilution) was from QED Bioscience, the antibody against GFP (ab290; 1:3000 dilution) was from Abcam, the antibody against PABPC1 (#4992; 1:3000 dilution) from Cell Signaling Technology, the antibody against tubulin (T6074; 1:3000 dilution) was from Sigma, and the antibody against UPF2 was kindly provided by Jens Lykke-Andersen (1:1000 dilution). Secondary horseradish peroxidase-coupled antibodies against rabbit (111-035-006; 1:3000 dilution) or mouse (115-035-003; 1:3000 dilution) were from Jackson ImmunoResearch. Western Lightning Plus-ECL Enhanced Chemiluminescence Substrate (PerkinElmer) in combination with the myECL Imager (ThermoFisher) was used for visualization.

## Additional Information

**How to cite this article**: Fatscher, T. and Gehring, N. H. Harnessing short poly(A)-binding protein-interacting peptides for the suppression of nonsense-mediated mRNA decay. *Sci. Rep.* **6**, 37311; doi: 10.1038/srep37311 (2016).

**Publisher’s note:** Springer Nature remains neutral with regard to jurisdictional claims in published maps and institutional affiliations.

## References

[b1] Chang Y. F., Imam J. S. & Wilkinson M. F. The nonsense-mediated decay RNA surveillance pathway. Annual review of biochemistry 76, 51–74, doi: 10.1146/annurev.biochem.76.050106.093909 (2007). 17352659

[b2] Nicholson P. . Nonsense-mediated mRNA decay in human cells: mechanistic insights, functions beyond quality control and the double-life of NMD factors. Cell Mol Life Sci 67, 677–700, doi: 10.1007/s00018-009-0177-1 (2010). 19859661 PMC11115722

[b3] Rebbapragada I. & Lykke-Andersen J. Execution of nonsense-mediated mRNA decay: what defines a substrate? Curr Opin Cell Biol 21, 394–402, doi: 10.1016/j.ceb.2009.02.007 (2009). 19359157

[b4] Thermann R. . Binary specification of nonsense codons by splicing and cytoplasmic translation. The EMBO journal 17, 3484–3494, doi: 10.1093/emboj/17.12.3484 (1998). 9628884 PMC1170685

[b5] Zhang J., Sun X. L., Qian Y. M. & Maquat L. E. Intron function in the nonsense-mediated decay of beta-globin mRNA: Indications that pre-mRNA splicing in the nucleus can influence mRNA translation in the cytoplasm. RNA 4, 801–815, doi: 10.1017/S1355838298971849 (1998). 9671053 PMC1369660

[b6] Le Hir H., Izaurralde E., Maquat L. E. & Moore M. J. The spliceosome deposits multiple proteins 20-24 nucleotides upstream of mRNA exon-exon junctions. The EMBO journal 19, 6860–6869, doi: 10.1093/emboj/19.24.6860 (2000). 11118221 PMC305905

[b7] Bhattacharya A. . Characterization of the biochemical properties of the human Upf1 gene product that is involved in nonsense-mediated mRNA decay. RNA 6, 1226–1235 (2000). 10999600 10.1017/s1355838200000546 PMC1369996

[b8] Chamieh H., Ballut L., Bonneau F. & Le Hir H. NMD factors UPF2 and UPF3 bridge UPF1 to the exon junction complex and stimulate its RNA helicase activity. Nat Struct Mol Biol 15, 85–93, doi: 10.1038/nsmb1330 (2008). 18066079

[b9] Leeds P., Peltz S. W., Jacobson A. & Culbertson M. R. The product of the yeast UPF1 gene is required for rapid turnover of mRNAs containing a premature translational termination codon. Genes Dev 5, 2303–2314 (1991). 1748286 10.1101/gad.5.12a.2303

[b10] Okada-Katsuhata Y. . N- and C-terminal Upf1 phosphorylations create binding platforms for SMG-6 and SMG-5:SMG-7 during NMD. Nucleic Acids Res 40, 1251–1266, doi: 10.1093/nar/gkr791 (2012). 21965535 PMC3273798

[b11] Tani H. . Identification of hundreds of novel UPF1 target transcripts by direct determination of whole transcriptome stability. RNA Biol 9, 1370–1379, doi: 10.4161/rna.22360 (2012). 23064114 PMC3597577

[b12] Yepiskoposyan H., Aeschimann F., Nilsson D., Okoniewski M. & Muhlemann O. Autoregulation of the nonsense-mediated mRNA decay pathway in human cells. RNA 17, 2108–2118, doi: 10.1261/rna.030247.111 (2011). 22028362 PMC3222124

[b13] Eberle A. B., Stalder L., Mathys H., Orozco R. Z. & Muhlemann O. Posttranscriptional gene regulation by spatial rearrangement of the 3′ untranslated region. Plos Biol 6, e92, doi: 10.1371/journal.pbio.0060092 (2008). 18447580 PMC2689704

[b14] Singh G., Rebbapragada I. & Lykke-Andersen J. A competition between stimulators and antagonists of Upf complex recruitment governs human nonsense-mediated mRNA decay. Plos Biol 6, e111, doi: 10.1371/journal.pbio.0060111 (2008). 18447585 PMC2689706

[b15] Boehm V., Haberman N., Ottens F., Ule J. & Gehring N. H. 3′ UTR length and messenger ribonucleoprotein composition determine endocleavage efficiencies at termination codons. Cell Rep 9, 555–568, doi: 10.1016/j.celrep.2014.09.012 (2014). 25310981

[b16] Toma K. G., Rebbapragada I., Durand S. & Lykke-Andersen J. Identification of elements in human long 3′ UTRs that inhibit nonsense-mediated decay. RNA 21, 887–897, doi: 10.1261/rna.048637.114 (2015). 25805855 PMC4408796

[b17] Fatscher T., Boehm V., Weiche B. & Gehring N. H. The interaction of cytoplasmic poly(A)-binding protein with eukaryotic initiation factor 4G suppresses nonsense-mediated mRNA decay. RNA 20, 1579–1592, doi: 10.1261/rna.044933.114 (2014). 25147240 PMC4174440

[b18] Joncourt R., Eberle A. B., Rufener S. C. & Muhlemann O. Eukaryotic initiation factor 4G suppresses nonsense-mediated mRNA decay by two genetically separable mechanisms. PLoS One 9, e104391, doi: 10.1371/journal.pone.0104391 (2014). 25148142 PMC4141738

[b19] Silva A. L., Ribeiro P., Inacio A., Liebhaber S. A. & Romao L. Proximity of the poly(A)-binding protein to a premature termination codon inhibits mammalian nonsense-mediated mRNA decay. RNA 14, 563–576, doi: 10.1261/rna.815108 (2008). 18230761 PMC2248256

[b20] Behm-Ansmant I., Gatfield D., Rehwinkel J., Hilgers V. & Izaurralde E. A conserved role for cytoplasmic poly(A)-binding protein 1 (PABPC1) in nonsense-mediated mRNA decay. The EMBO journal 26, 1591–1601, doi: 10.1038/sj.emboj.7601588 (2007). 17318186 PMC1829367

[b21] Kozlov G. . Structural basis of ligand recognition by PABC, a highly specific peptide-binding domain found in poly(A)-binding protein and a HECT ubiquitin ligase. The EMBO journal 23, 272–281, doi: 10.1038/sj.emboj.7600048 (2004). 14685257 PMC1271756

[b22] Kozlov G. & Gehring K. Molecular basis of eRF3 recognition by the MLLE domain of poly(A)-binding protein. PLoS One 5, e10169, doi: 10.1371/journal.pone.0010169 (2010). 20418951 PMC2854688

[b23] Kozlov G. . Structure and function of the C-terminal PABC domain of human poly(A)-binding protein. Proceedings of the National Academy of Sciences of the United States of America 98, 4409–4413, doi: 10.1073/pnas.071024998 (2001). 11287632 PMC31848

[b24] Albrecht M. & Lengauer T. Survey on the PABC recognition motif PAM2. Biochem Biophys Res Commun 316, 129–138, doi: 10.1016/j.bbrc.2004.02.024 (2004). 15003521

[b25] Hoshino S., Imai M., Kobayashi T., Uchida N. & Katada T. The eukaryotic polypeptide chain releasing factor (eRF3/GSPT) carrying the translation termination signal to the 3′-Poly(A) tail of mRNA. Direct association of erf3/GSPT with polyadenylate-binding protein. J Biol Chem 274, 16677–16680, doi: 10.1074/jbc.274.24.16677 (1999). 10358005

[b26] Uchida N., Hoshino S., Imataka H., Sonenberg N. & Katada T. A novel role of the mammalian GSPT/eRF3 associating with poly(A)-binding protein in Cap/Poly(A)-dependent translation. J Biol Chem 277, 50286–50292, doi: 10.1074/jbc.M203029200 (2002). 12381739

[b27] Siddiqui N. . Poly(A) nuclease interacts with the C-terminal domain of polyadenylate-binding protein domain from poly(A)-binding protein. J Biol Chem 282, 25067–25075, doi: 10.1074/jbc.M701256200 (2007). 17595167

[b28] Ezzeddine N. . Human TOB, an antiproliferative transcription factor, is a poly(A)-binding protein-dependent positive regulator of cytoplasmic mRNA deadenylation. Mol Cell Biol 27, 7791–7801, doi: 10.1128/MCB.01254-07 (2007). 17785442 PMC2169145

[b29] Khaleghpour K. . Translational repression by a novel partner of human poly(A) binding protein, Paip2. Molecular cell 7, 205–216 (2001). 11172725 10.1016/s1097-2765(01)00168-x

[b30] Bhuvanagiri M., Schlitter A. M., Hentze M. W. & Kulozik A. E. NMD: RNA biology meets human genetic medicine. Biochem J 430, 365–377, doi: 10.1042/BJ20100699 (2010). 20795950

[b31] Culbertson M. R. RNA surveillance. Unforeseen consequences for gene expression, inherited genetic disorders and cancer. Trends Genet 15, 74–80 (1999). 10098411 10.1016/s0168-9525(98)01658-8

[b32] Peltz S. W., Morsy M., Welch E. M. & Jacobson A. Ataluren as an agent for therapeutic nonsense suppression. Annu Rev Med 64, 407–425, doi: 10.1146/annurev-med-120611-144851 (2013). 23215857 PMC3968684

[b33] Kerr T. P., Sewry C. A., Robb S. A. & Roberts R. G. Long mutant dystrophins and variable phenotypes: evasion of nonsense-mediated decay? Hum Genet 109, 402–407, doi: 10.1007/s004390100598 (2001). 11702221

[b34] Safaee N. . Interdomain allostery promotes assembly of the poly(A) mRNA complex with PABP and eIF4G. Molecular cell 48, 375–386, doi: 10.1016/j.molcel.2012.09.001 (2012). 23041282

[b35] Tarun S. Z. Jr. & Sachs A. B. Association of the yeast poly(A) tail binding protein with translation initiation factor eIF-4G. The EMBO journal 15, 7168–7177 (1996). 9003792 PMC452544

[b36] Wells S. E., Hillner P. E., Vale R. D. & Sachs A. B. Circularization of mRNA by eukaryotic translation initiation factors. Molecular cell 2, 135–140 (1998). 9702200 10.1016/s1097-2765(00)80122-7

[b37] Thompson K. E., Bashor C. J., Lim W. A. & Keating A. E. SYNZIP protein interaction toolbox: *in vitro* and *in vivo* specifications of heterospecific coiled-coil interaction domains. ACS Synth Biol 1, 118–129, doi: 10.1021/sb200015u (2012). 22558529 PMC3339576

[b38] Kononenko A. V. . GTP-dependent structural rearrangement of the eRF1:eRF3 complex and eRF3 sequence motifs essential for PABP binding. Nucleic Acids Res 38, 548–558, doi: 10.1093/nar/gkp908 (2010). 19906736 PMC2811017

[b39] Osawa M. . Biological role of the two overlapping poly(A)-binding protein interacting motifs 2 (PAM2) of eukaryotic releasing factor eRF3 in mRNA decay. RNA 18, 1957–1967, doi: 10.1261/rna.035311.112 (2012). 23019593 PMC3479387

[b40] Kahvejian A., Svitkin Y. V., Sukarieh R., M’Boutchou M. N. & Sonenberg N. Mammalian poly(A)-binding protein is a eukaryotic translation initiation factor, which acts via multiple mechanisms. Genes Dev 19, 104–113, doi: 10.1101/gad.1262905 (2005). 15630022 PMC540229

[b41] Lykke-Andersen J., Shu M. D. & Steitz J. A. Communication of the position of exon-exon junctions to the mRNA surveillance machinery by the protein RNPS1. Science 293, 1836–1839, doi: 10.1126/science.1062786 (2001). 11546874

[b42] Mayeda A. . Purification and characterization of human RNPS1: a general activator of pre-mRNA splicing. The EMBO journal 18, 4560–4570, doi: 10.1093/emboj/18.16.4560 (1999). 10449421 PMC1171530

[b43] Gehring N. H., Lamprinaki S., Hentze M. W. & Kulozik A. E. The hierarchy of exon-junction complex assembly by the spliceosome explains key features of mammalian nonsense-mediated mRNA decay. Plos Biol 7, e1000120, doi: 10.1371/journal.pbio.1000120 (2009). 19478851 PMC2682485

[b44] Baserga S. J. & Benz E. J. Jr. Nonsense mutations in the human beta-globin gene affect mRNA metabolism. Proceedings of the National Academy of Sciences of the United States of America 85, 2056–2060 (1988). 3353367 10.1073/pnas.85.7.2056 PMC279927

[b45] Pergolizzi R. . Two cloned beta thalassemia genes are associated with amber mutations at codon 39. Nucleic acids research 9, 7065–7072 (1981). 6278453 10.1093/nar/9.24.7065 PMC327662

[b46] Ge Z., Quek B. L., Beemon K. L. & Hogg J. R. Polypyrimidine tract binding protein 1 protects mRNAs from recognition by the nonsense-mediated mRNA decay pathway. Elife 5, doi: 10.7554/eLife.11155 (2016). PMC4764554 26744779

[b47] Lee S. R., Pratt G. A., Martinez F. J., Yeo G. W. & Lykke-Andersen J. Target Discrimination in Nonsense-Mediated mRNA Decay Requires Upf1 ATPase Activity. Molecular cell 59, 413–425, doi: 10.1016/j.molcel.2015.06.036 (2015). 26253027 PMC4673969

[b48] Callaghan M. J. . Identification of a human HECT family protein with homology to the Drosophila tumor suppressor gene hyperplastic discs. Oncogene 17, 3479–3491, doi: 10.1038/sj.onc.1202249 (1998). 10030672

[b49] Deo R. C., Sonenberg N. & Burley S. K. X-ray structure of the human hyperplastic discs protein: an ortholog of the C-terminal domain of poly(A)-binding protein. Proceedings of the National Academy of Sciences of the United States of America 98, 4414–4419, doi: 10.1073/pnas.071552198 (2001). 11287654 PMC31849

[b50] Adamala K. P., Martin-Alarcon D. A. & Boyden E. S. Programmable RNA-binding protein composed of repeats of a single modular unit. Proceedings of the National Academy of Sciences of the United States of America 113, E2579–E2588, doi: 10.1073/pnas.1519368113 (2016). 27118836 PMC4868411

[b51] Porter D. F., Koh Y. Y., VanVeller B., Raines R. T. & Wickens M. Target selection by natural and redesigned PUF proteins. Proceedings of the National Academy of Sciences of the United States of America 112, 15868–15873, doi: 10.1073/pnas.1508501112 (2015). 26668354 PMC4703012

[b52] Nelles D. A. . Programmable RNA Tracking in Live Cells with CRISPR/Cas9. Cell 165, 488–496, doi: 10.1016/j.cell.2016.02.054 (2016). 26997482 PMC4826288

[b53] Price A. A., Sampson T. R., Ratner H. K., Grakoui A. & Weiss D. S. Cas9-mediated targeting of viral RNA in eukaryotic cells. Proceedings of the National Academy of Sciences of the United States of America 112, 6164–6169, doi: 10.1073/pnas.1422340112 (2015). 25918406 PMC4434742

[b54] Nomakuchi T. T., Rigo F., Aznarez I. & Krainer A. R. Antisense oligonucleotide-directed inhibition of nonsense-mediated mRNA decay. Nat Biotechnol 34, 164–166, doi: 10.1038/nbt.3427 (2016). 26655495 PMC4744113

[b55] Gehring N. H. . Exon-junction complex components specify distinct routes of nonsense-mediated mRNA decay with differential cofactor requirements. Molecular cell 20, 65–75, doi: 10.1016/j.molcel.2005.08.012 (2005). 16209946

[b56] Gehring N. H., Lamprinaki S., Kulozik A. E. & Hentze M. W. Disassembly of exon junction complexes by PYM. Cell 137, 536–548, doi: 10.1016/j.cell.2009.02.042 (2009). 19410547

[b57] Steckelberg A. L., Boehm V., Gromadzka A. M. & Gehring N. H. CWC22 connects pre-mRNA splicing and exon junction complex assembly. Cell reports 2, 454–461, doi: 10.1016/j.celrep.2012.08.017 (2012). 22959432

[b58] Ivanov P. V., Gehring N. H., Kunz J. B., Hentze M. W. & Kulozik A. E. Interactions between UPF1, eRFs, PABP and the exon junction complex suggest an integrated model for mammalian NMD pathways. The EMBO journal 27, 736–747, doi: 10.1038/emboj.2008.17 (2008). 18256688 PMC2265754

[b59] Hundsdoerfer P., Thoma C. & Hentze M. W. Eukaryotic translation initiation factor 4GI and p97 promote cellular internal ribosome entry sequence-driven translation. Proceedings of the National Academy of Sciences of the United States of America 102, 13421–13426, doi: 10.1073/pnas.0506536102 (2005). 16174738 PMC1224658

